# ClearFinder: a Python GUI for annotating cells in cleared mouse brain

**DOI:** 10.1186/s12859-025-06039-x

**Published:** 2025-01-21

**Authors:** Stefan Pastore, Philipp Hillenbrand, Nils Molnar, Irina Kovlyagina, Monika Chanu Chongtham, Stanislav Sys, Beat Lutz, Margarita Tevosian, Susanne Gerber

**Affiliations:** 1 Institute for Human Genetics, University Medical Center Johannes Gutenberg University, 55131 Mainz, Germany; 2 Institute of Pharmaceutical and Biomedical Sciences, Johannes Gutenberg University, 55128 Mainz, Germany; 3 Institute of Physiological Chemistry, University Medical Center Johannes Gutenberg University, 55128 Mainz, Germany; 4 Leibniz Institute for Resilience Research, 55122 Mainz, Germany

**Keywords:** Tissue clearing, Atlas alignment, Cell count, 3D volumetric imaging

## Abstract

**Background:**

Tissue clearing combined with light-sheet microscopy is gaining popularity among neuroscientists interested in unbiased assessment of their samples in 3D volume. However, the analysis of such data remains a challenge. ClearMap and CellFinder are tools for analyzing neuronal activity maps in an intact volume of cleared mouse brains. However, these tools lack a user interface, restricting accessibility primarily to scientists proficient in advanced Python programming. The application presented here aims to bridge this gap and make data analysis accessible to a wider scientific community.

**Results:**

We developed an easy-to-adopt graphical user interface for cell quantification and group analysis of whole cleared adult mouse brains. Fundamental statistical analysis, such as PCA and box plots, and additional visualization features allow for quick data evaluation and quality checks. Furthermore, we present a use case of ClearFinder GUI for cross-analyzing the same samples with two cell counting tools, highlighting the discrepancies in cell detection efficiency between them.

**Conclusions:**

Our easily accessible tool allows more researchers to implement the methodology, troubleshoot arising issues, and develop quality checks, benchmarking, and standardized analysis pipelines for cell detection and region annotation in whole volumes of cleared brains.

**Supplementary Information:**

The online version contains supplementary material available at 10.1186/s12859-025-06039-x.

## Background

Immediate-early genes (IEGs) expression has been used for decades to visualize populations of activated neurons. Nowadays, neuroscientists frequently use activity-dependent promoters and transgenic reporter mouse lines for permanent labelling of neurons at selected time points [1]. However, to detect fluorescently tagged ensembles of neurons distributed throughout the brain, the tissue has to be sectioned, resulting in bias and artifacts. A new approach to overcome these limitations was recently developed: tissue clearing combined with light-sheet fluorescence microscopy. This concept gained popularity among neuroscientists studying the complexity of the nervous system [2].

One of the major applications of this methodology is to identify the brain regions where neuronal activity is down- or upregulated. This is achieved by first detecting individual cells in a 3D volume and then performing a non-linear transformation of the images to the reference atlas. However, the acquisition of the complete volume of a cleared adult mouse brain can result in several terabytes of data, necessitating a sophisticated infrastructure with high data storage capacity and powerful computational resources for processing.

Currently, ClearMap [3, 4] and CellFinder [5] are the only open-source tools for cell detection and annotation in whole/intact brain volume. However, both software solutions—albeit very useful—are command-line based and require third-party packages that are frequently not maintained. In addition, neither tool offers statistical analysis and data visualization functionality. Despite some attempts at making these pipelines more accessible to biologists and microscopists [6], the installation and usage still require intensive support from skilled bioinformaticians or computer scientists.

Here, we developed ClearFinder, a Graphical User Interface (GUI) unifying the functionality of both ClearMap and CellFinder, while at the same time simplifying the setup process and offering fundamental statistical analysis of results. The user is guided through streamlined data selection and preparation steps, including uniform file renaming, adjustment of cell detection parameters and image pre-processing, alignment to a reference atlas, and generation of an output file containing cell counts per brain region. To showcase the utility of ClearFinder, we analyzed three brain samples with both sub-packages: ClearMap and CellFinder. The results are also visualized using the GUI functionality, illustrating the benefits of a user interface for a potential application.

## Materials and methods

### Animals

All experiments were performed according to the European Community's Council Directive of 22 September 2010 (2010/63EU) and approved by the respective agency of the State Rhineland-Palatinate (Landesuntersuchungsamt, permit number G-17-1-021). The animals were bred at the Translational Animal Research Center (TARC) in Mainz for the Leibniz Institute for Resilience Research.

Male mice were group-housed in temperature- and humidity-controlled rooms with a 12-h light–dark cycle with water and food provided ad libitum. Seven days prior to experiments, animals were single-housed. Mice used in this study were 8 weeks old by the start of the experiments. B6.Cg-Tg(Arc-cre/ERT2)MRhn/CdnyJ mice (JAX Nr. 022357 [7]) were crossed with B6.129-Gt(ROSA)26Sortm5(CAG-Sun1/sfGFP)Nat/J mice [8], and generated double transgenic heterozygous mice are referred to as Arc-nuclGFP for simplicity. Upon neuronal activity, in the presence of tamoxifen (Merck, Germany), neurons in these mice can express a nuclear membrane variant of green fluorescent protein (Sun1/sfGFP), thereby obtaining permanent labelling of these cells. Mice received an intraperitoneal (i.p.) injection of tamoxifen (150 mg/kg) during chronic social defeat stress paradigm [9], as described below. Afterwards, mice were deeply anesthetized by i.p. injection of 100 mg/kg pentobarbital (Merck, Germany) and 0.1 mg/kg buprenorphine (TEMGESIC, Indivior Europe Ltd, Ireland) in water. After checking for the absence of the interdigital reflex, mice were perfused transcardially with 100 ml ice-cold phosphate-buffered saline (PBS), pH 7.8, and 50 ml 4% freshly prepared paraformaldehyde (PFA) solution. The brain was dissected and used for further sample preparation.

### Chronic social defeat

Chronic social defeat (CSD) stress was carried out as previously reported [9]. Briefly, experimental mice and adult retired breeders CD1 mice (Charles River) were housed in the same cage but were physically separated by a perforated metal grid for 10 days. During this period, the separating grid was removed every day for 15 s three times per day to allow physical attack from CD1 mice. After 10 days of CSD, mice were allowed to rest for 1 week (no exposure to CD1, single housing in the home cage).

### Sample preparation

The samples were post-fixed in 4% PFA at 4 °C for 8 h, then washed in PBS 3 times for 30 min each.

iDISCO + tissue clearing was performed according to Jin et al. [10] with modifications. Briefly, dissected tissue was washed in PBS and dehydrated at room temperature (RT) in MeOH (Merck, Germany) solutions in water: 20, 40, 60, 80, 100% MeOH for 1 h each, followed by incubating in 100% MeOH overnight at RT. Afterwards, tissue delipidation was performed by incubating the samples in dichloromethane (DCM; Merck, Germany) and MeOH solution (1:3) twice for 2 h at RT. After washing the samples for 1 h in 100% MeOH, they were transferred to 5% H_2_O_2_ (Merck, Germany) in 100% MeOH solution overnight at 4 °C. After rehydrating the samples in decreasing concentrations of MeOH solution in water at RT (80, 60, 40, 20% MeOH for 1 h each), they were transferred to permeabilization solution (78.6% PTx0.5 (PBS with 0.5% TritonX-100); 1.4% glycine; 20% dimethyl sulfoxide, DMSO) for 2 days at 37 °C, followed by blocking solution (84% PTx0.5; 6% normal donkey serum, NDS; 10% DMSO) for 2 days at 37 °C. Afterwards, the samples were incubated at 37 °C in 1:1000 diluted primary antibody (chicken anti-GFP, Aves Labs, Davies, CA, USA; RRID:AB_10000240) solution (92% PTwH0.5 (PBS with 0.5% Tween 20 and 10 mg/ml heparin); 3% NDS; 5% DMSO) for 7 days, with sequential additions of the primary antibody on days 2, 3, 4 and 5, thereby reaching a final dilution of 1:200. The samples were washed in PTwH0.5 over 4 days at 37 °C and then incubated with the 1:500 diluted secondary antibody (Alexa Fluor® 647 AffiniPure F(ab')₂ Fragment Donkey Anti-Chicken IgG (H + L), Jackson ImmunoResearch, West Grove, PA, USA) solution (92% PTwH0.5; 3% NDS; 5% DMSO) for 7 days at 37 °C with sequential additions of the antibody on days 2, 3, 4 and 5, thereby reaching a final dilution of 1:100. The samples were washed in PTwH0.5 over 4 days at 37 °C. Then, the samples were dehydrated and delipidated again, as described above. Afterwards, they were incubated in dibenzyl ether (DBE; Sigma-Aldrich, Germany) overnight at RT for refractive index (RI) matching until fully transparent. Samples were stored and imaged in DBE at RT. All incubation procedures were performed under constant gentle mixing on a nutating mixer (ThermoFisher Scientific, USA).

Microscopy of cleared samples was performed in horizontal orientation on the light-sheet microscope UltraMicroscope II (Miltenyi Biotec) with a camera (Andor Neo sCMOS) and a 2x/0.5NA objective (MV PLAPO 2XC, Olympus) with corrected dipping cap attached. Zoom factor was set to × 1.6. Light sheet width was set to 70%, sheet NA—0.163 (3.9 µm thickness), merging algorithm—fixed blend, step size—3 µm, dynamic horizontal focus was used with 8 steps and fixed blending mode. Laser Module Beam combiner was used with separate laser channels: Ex: 640 nm, Em: 680 nm to visualize the fluorescent signal and Ex: 488 nm, Em: 525 nm to capture the autofluorescence of the tissue for later reference brain registration. Exposure time was set to 150 ms, sheet width to 100%, light-sheet merging algorithm—blend. Tile scan consisted of 6 tiles (2 in x and 3 in y axis) with an overlap between tiles of 20%. Acquired images were processed using Vision4D (Carl Zeiss Microscopy Software Center Rostock GmbH, Germany): tile stitching was performed after automatic tile alignment to compensate for stage positioning artefacts during imaging.

### Data analysis

All data analysis and plots were generated with the embedded functionality of the ClearFinder GUI: PCA, heatmaps; except for the ANOVA analysis from Fig. 3B. The software GraphPad Prism V6 (La Jolla, USA) was used for the ANOVA test, variability calculation, and the graphical visualization. Supplementary Fig. 4 was generated in Python using the Seaborn library.

### System requirements

Recommended PC requirements

OS: Linux (Debian Ubuntu)

CPU: Intel(R) Xeon(R) Silver 4210R CPU @ 2.40 GHz

Processors: 20 (Threads)

GPU: NVIDIA RTX A4000 @ 16 GB VRAM GDDR6

RAM: 128 GB

### Implementation

**Fig. 1:**
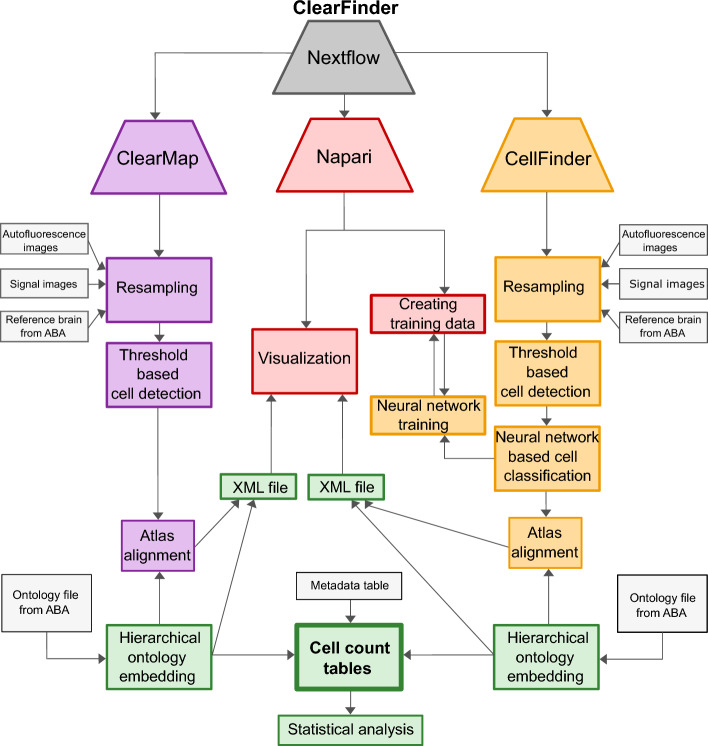
ClearFinder GUI architecture. Schematic representation of the workflow, unifying both analysis tools under one GUI. Trapezoids indicate individual software packages: violet—ClearMap, red—Napari, orange—CellFinder, grey—Nextflow. Separate analysis steps with each package are indicated in rectangular boxes of respective colors. Light-grey boxes indicate the input from the user. Green boxes indicate the output of ClearFinder GUI. ABA—Allen Brain Atlas

ClearFinder is written in Python using the QT framework. The integration of Napari [11], CellFinder, and ClearMap environments management, as well as job scheduling is orchestrated by Nextflow [12]. The workflow (Fig. 1) is directly applicable after the collection of TIFF files from stitched images from the light-sheet microscopy experiment. We enabled the uniform data output from 2 sub-packages, whereby the detected cells of ClearMap are converted into Extensible Markup Language (XML) format, enabling a CellFinder-like cell visualization in Napari.

The ClearFinder GUI additionally allows the training of CellFinder’s neuronal network for cell classification. Training data can be obtained with a CellFinder plugin for Napari.

Detected cells are embedded into a comparable data structure. The latter allows for a new feature compared to the original published algorithms: a summary of cell counts over different structural hierarchies based on the phylogeny of the Allen Brain Atlas (http://alleninstitute.org/) mouse ontology file.

**Fig. 2:**
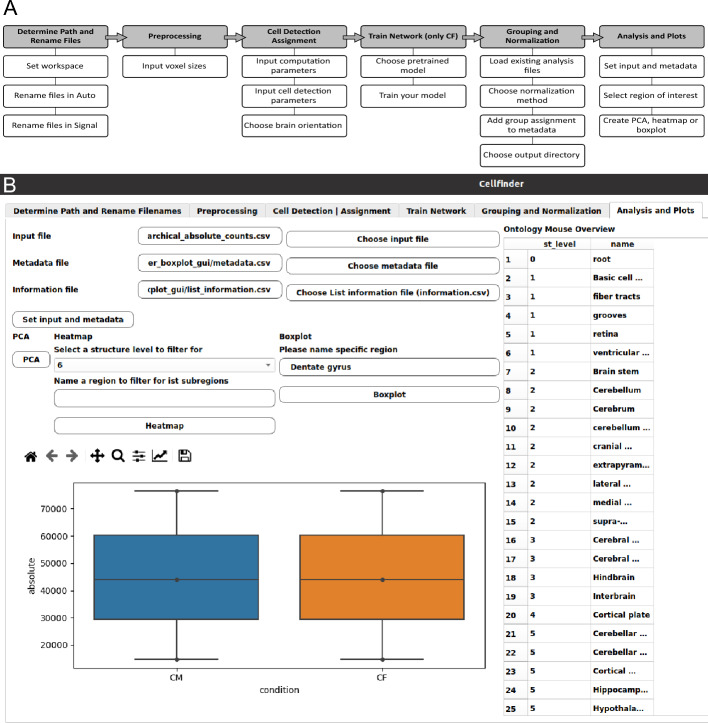
ClearFinder GUI interface. **A** A scheme illustrating a step-by-step process of loading, processing and analysis of the data, orchestrated by the ClearFinder GUI. Top row represents the major processing steps, including the selection options in black boxes below. **B** A screenshot of the GUI interface depicting the last step of the GUI wizard allowing basic analysis and visualization of obtained results

Cell counts of several samples can be integrated into a single data frame with an additional option to create a metadata frame, allocating the samples to respective experimental groups. Furthermore, two normalization options for the number of cells detected across different samples and experimental conditions are available: median of ratios [13] or cells per million (Fig. 2). A unified data output format allows a standardized statistical analysis between conditions across both tools.

CellFinder, ClearMap, and Napari are embedded in independent Conda environments and are available on GitHub (https://github.com/stegiopast/ClearFinder). Anaconda environments are an essential feature of the overall software design due to their practical ability to manage Python environments while simultaneously allowing the installation of required non-Python dependencies, such as CUDA, which are difficult to manage manually. Furthermore, placing CellFinder, Napari, and ClearMap in different environments allows the integration of tools into one whole software suite while keeping dependencies. Overall, this strategy guarantees future stability.

## Results

### ClearFinder GUI for neuronal activity mapping

Although both ClearMap and CellFinder are potent tools for cell detection in cleared tissue [14–18], both programs are quite challenging in terms of installation, data handling, setting the parameters for cell detection, and image transformation. These steps require manual execution via the command line.

To address these limitations, we developed a unified, intuitive interface: ClearFinder (Figs. 1, 2). The GUI offers several advantages, including streamlined setup for Ubuntu-based systems, renaming images and setting workspaces to process the gathered data. Subsequently, processing parameters for the resampling and cell detection steps can be adjusted easily (Fig. 2).

Moreover, ClearFinder integrates both ClearMap and CellFinder (as sub-packages) in a single toolbox by a Nextflow script workflow (Fig. 1). As a result, users can initiate both tools with a single bash command. Following independent processing with ClearMap and CellFinder sub-packages, the generated results can be easily visualized and compared in Napari, providing a cohesive data output for subsequent in-depth statistical analysis.

### ClearFinder reveals differences in total cell counts between ClearMap and CellFinder

**Fig. 3:**
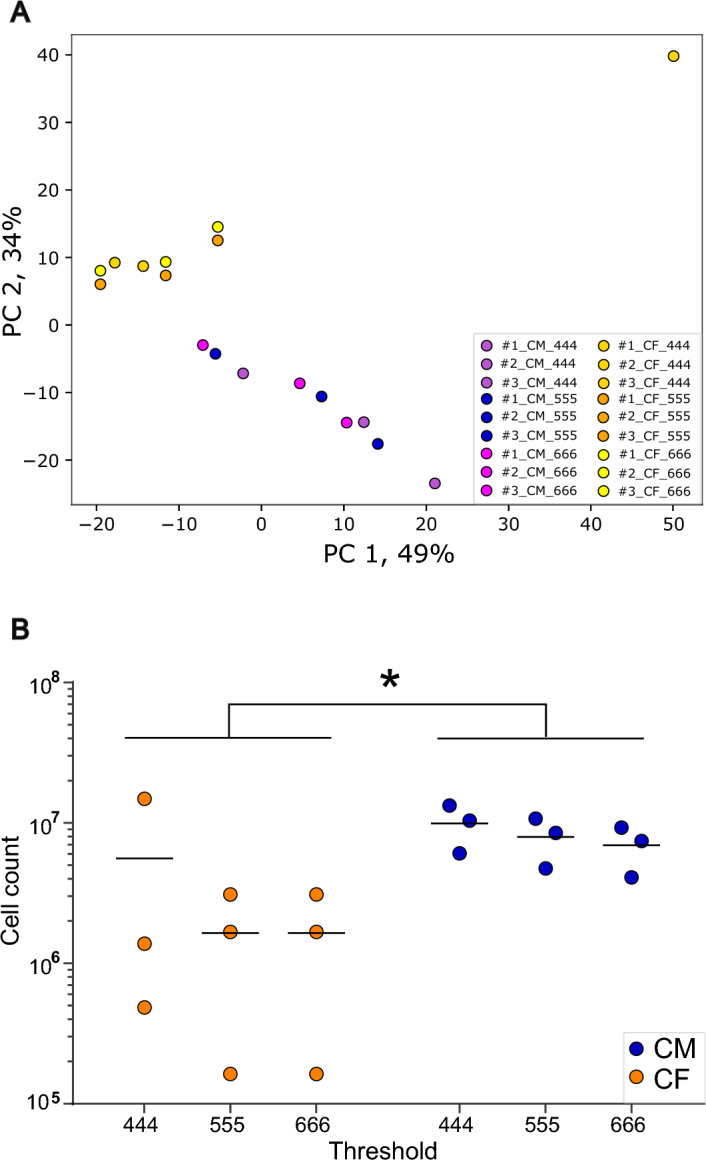
Differences in total cell counts between ClearMap and CellFinder. PCA plot of all cell counts in/across all brain regions. GFP-positive cells were detected in all regions of three sample using two methods and three detection thresholds. Yellow, orange and dark-orange dots indicate data obtained with CellFinder (CF), magenta, blue and violet dots indicate data processed with ClearMap (CM). CF dots were displaced by 2% vertically to avoid a complete overlap. PC—principal component. **B** Total cell counts across all regions. Total counts of GFP-positive cells detected across all brain regions using two methods and three detection thresholds. The y axis represents the log-transformed cell counts. Significant differences between methods identified by 2-way ANOVA, p_method_ = 0.0165, p_threshold_ = 0.3131, p_method×threshold_ = 0.3131. *p < 0.05. CF—CellFinder, CM—ClearMap

To demonstrate the utility and functionality of the ClearFinder GUI, we analyzed one dataset (n = 3). To this end, Arc-nuclGFP [7, 8] (see “Materials and methods” section) mice were injected with tamoxifen after chronic social defeat to induce permanent GFP labelling of neuronal nuclei throughout the brain. Later, the brain tissue was dissected, stained, cleared using iDISCO + and imaged on a light-sheet microscope (Ultramicroscope II) in intact volume. After initial preprocessing (tile scan stitching using commercial software arivis Vision 4D), the data was analyzed using ClearFinder. We performed cell detection with three different size-thresholds (4, 5, and 6 µm in three axes), then the cells were allocated to the mouse Allen Brain Atlas [19]. The principal component analysis (PCA, Fig. 3A) conducted on the total number of GFP-positive cells across all brain regions unveiled considerable disparities between the sub-packages and thresholds. Notably, we detected high variability among samples subjected to cross-analysis using identical thresholds but different methods. In a 2-way ANOVA, total cell counts significantly differed between detection methods, but not thresholds (Fig. 3B). The variability was calculated as coefficient of variation (CV), also known as “relative variability”, which equals the standard deviation divided by the mean. We selected the threshold value of 555 (5 µm in each axis) for further analysis since it produced results with the lowest within-group variability for the CellFinder sub-package (CV = 89.093 vs. 89.097 and 144.454 for thresholds 666 and 444 respectively); while the variability between the samples analyzed with the ClearMap sub-package was comparable for all three thresholds (CV = 36.610, 37.89338, and 37.81342 for thresholds 444, 555, 666 respectively).

### ClearFinder reveals discrepancies in cell detection efficiency

**Fig. 4:**
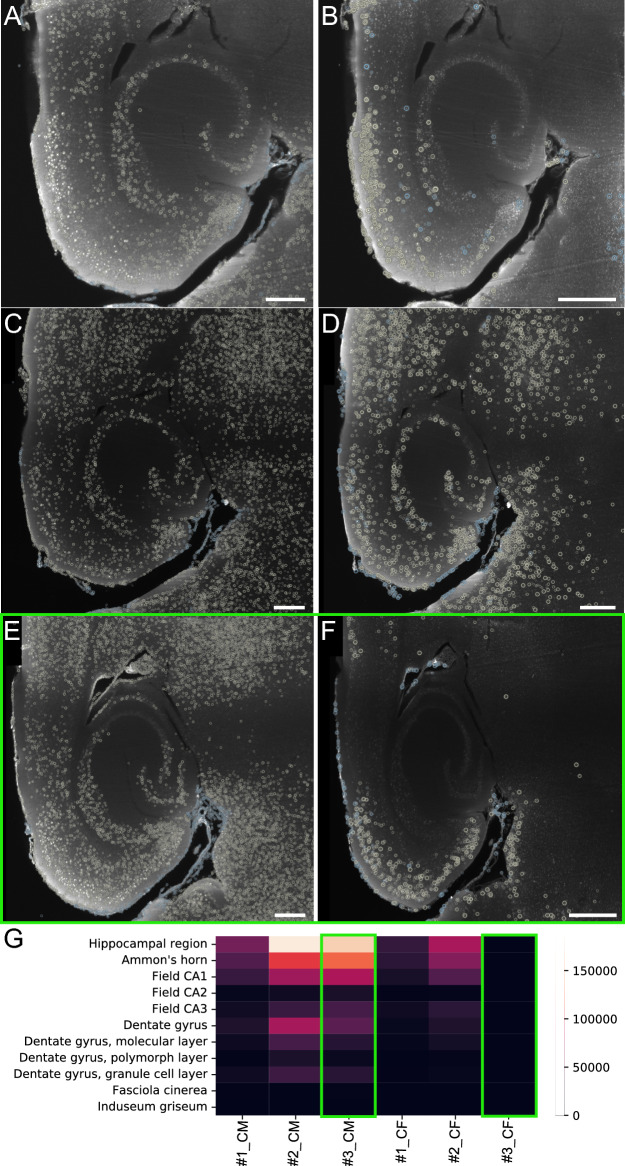
Cell detection efficiency. Sample #1 and Sample #2 analyzed with ClearMap (**A**, **C**) and CellFinder (**B**, **D**) using threshold 555. Sample #3 analyzed with ClearMap (**E**) and CellFinder (**F**) using threshold 555, marked by a green rectangle. Yellow circles indicate cell maxima: “cells”. Blue circles indicate maxima unassigned to a brain region: “non-cells”. The radius of the cells indicates the proximity to the cell core in the selected optical plane. Smaller circles indicate the cell maxima out of plane. Scale bar: 150 µm. **G** Heatmap indicating the number of cells detected in subregions of the hippocampal area, as detected in each sample by ClearMap (CM) and CellFinder (CF) using the same threshold. Sample #3 marked by a green rectangle. Color-bar corresponds to cell count

The brain regions annotated in the Allen Brain Atlas are organized in 12 ontological levels from 0 being the root, containing all other brain regions, to 12, encapsulating the smallest subregions and nuclei. ClearFinder allows the sub-selection of ontological levels for easy analysis of a region of interest. We selected the hippocampal region (ontological level 6) for a side-by-side visual inspection of cell detection. The cells were detected using ClearMap (Fig. 4A, C, E) and CellFinder (Fig. 4B, D, F), and visualized in Napari. The comparison further highlighted noticeable differences in cell detection between the two sub-packages. Noteworthy, the performance of CellFinder was not consistent. CellFinder detected fewer cells in sample #3 (Fig. 4F), compared to ClearMap (Fig. 4E), while a comparable number of cells were detected in sample #2 (Fig. 4C, D). These prominent differences cannot be attributed to the additional machine learning-based cell/non-cell discrimination. Similar to ClearMap, the cell detection algorithm of CellFinder is based on threshold segmentation. Subsequent to cell detection, CellFinder includes a binary classification of detected maxima into cells and non-cells. Since the number of non-cells was negligible in analyzed samples (blue circles in Fig. 4E, F), the classification step was not contributing to the observed differences in cell counts Further, we visualized cell counts across the subregions of the hippocampal area (Fig. 4G), as well as a selection of cortical (Suppl. Fig. 1) and subcortical regions (Suppl. Fig. 2) as heatmaps. We observed consistently higher cell counts in all samples analyzed with ClearMap than with the CellFinder sub-package of ClearFinder GUI.

In summary, ClearFinder facilitates cell detection in cleared mouse brains with both ClearMap and CellFinder sub-packages in a unified GUI-assisted pipeline. Integrated features also enable visualization and statistical analysis of results. Side-by-side comparison of cell counts obtained with both ClearMap and CellFinder allows users to focus on samples with consistent results irrespective of the detection strategy, highlighting the need for appropriate sample size in biological studies. This validation process enhances the robustness and biological validity of the findings.

### Validation of cell detection efficiency of ClearFinder sub-packages by comparing to human annotation

To further elaborate on the inconsistencies in the performance of cell detection algorithms, we subsampled whole brain images into 18 randomly selected 60 µm^3^ cubes (Suppl. Fig. 3) and compared the cell detection efficiency between human experts and ClearFinder sub-packages. Two human experts, blinded to the sample identifiers, quantified the cells in three-dimensional space. Together, they identified 305 cells, achieving a 74.2% overlap in their annotations (Suppl. Fig. 4A). Subsequently, we compared human counts to those obtained from the ClearFinder sub-packages using the threshold 555 (Suppl. Fig. 4B–D). ClearMap detection performance reached 80.40% ± 21.49% (234/305 cells), whereas Cellfinder performance was 38.32% ± 40.39% (87/305 cells). Additionally, we observed between-sample variability in cell detection accuracy as compared to human count. Namely, ClearMap detected 83.04% ± 16.49% for sample #1, 74.52% ± 33.79% for sample #2, and 83.36% ± 9.69% for sample #3, while Cellfinder detected respectively 58.50% ± 23.12%, 40.39% ± 34.53% and 16.07% ± 25.33%, of the cells annotated by human experts (Suppl. Fig. 4E). These observations are in line with the results reported in Fig. 4.

## Documentation

An extensive documentation and the code are available on the project's GitHub (https://github.com/stegiopast/ClearFinder). We provide instructions on how to set up the workflow using Conda environments. ClearFinder targets Linux systems and can access other operating systems using a virtual machine.

## Conclusions

ClearFinder offers a comprehensive and user-friendly solution tailored for scientists from various disciplines to process whole-brain light-sheet microscopy imaging data efficiently. Key features include:

- *Intuitive interface:* ClearFinder provides a guided platform for detecting and assigning cells throughout the entire mouse brain volume using the capabilities of the ClearMap and CellFinder sub-packages
- *Robust installation process:* by maintaining independent virtual environments, ClearFinder ensures a robust and standardized installation process, simplifying the setup for users
- *Enhanced functionality* ClearFinder extends basic processing capabilities by integrating additional features such as heatmap visualization and fundamental statistical analysis tools, including PCA and box plots. It offers two options for cell count normalization facilitating further group comparisons. The unified XML data output from both sub-packages allows side-by-side visualization in Napari. Moreover, the ability to assign cell counts to different structural levels, along with the standardized output data formats from ClearFinder’s sub-packages, simplifies the process of conducting comprehensive comparative analyses across datasets.

Moreover, using ClearFinder to cross-analyze a dataset, we demonstrated discrepancies in cell detection performance, raising the reproducibility issue within the field. Our tool allows the researchers to visually inspect the quality of cell detection, visualize the results and focus on consistent data from both analysis pipelines. In our view, the cross-analysis of intact whole brain samples with two algorithms delivers a more robust and unbiased quantification, as compared to human annotation limited to selected sub-regions. We believe that our user-friendly ClearFinder GUI will encourage greater adoption of neuronal mapping tools, advancing the field, and leading to continuous improvement of the quality and reliability of these analysis tools.

## Supplementary Information

Supplementary material 1: Figure 1. Cell detection efficiency in cortical regions. Heatmap indicating the number of cells detected in the subregions of the anterior cingulate, prelimbic, and infralimbic areas as detected in each sample by ClearMap (CM) and CellFinder (CF) using the same threshold. Color-bar corresponds to cell count. Regions with no cell counts were not accounted in the heatmap

Supplementary material 2: Figure 2. Cell detection efficiency in subcortical regions. Heatmap indicating the number of cells detected in the subregions of the central, medial, lateral, basolateral, basomedial, and posterior amygdalar nuclei as detected in each sample by ClearMap (CM) and CellFinder (CF) using the same threshold. Color-bar corresponds to cell count. Regions with no cell counts were not accounted in the heatmap

Supplementary material 3: Figure 3. Image cubes for cell detection performance assessment. Examples of image cubes of sample #1 (**A**), #2 (**B**) and #3 (**C**). White dots mark counted cells of expert 1, red dots of expert 2, yellow circles of ClearMap and green circles of CellFinder. For sample collection 18 randomly selected x,y and z coordinates were generated. With these start coordinates cubes with a volume of 60µm^3^ of the raw signal files were created using ImageJ. Due to inevitable resizing events during the Cell detection process with CellFinder, the coordinates had to be re-transformed to the original signal image sizes. The latter was performed by multiplying the determined coordinates with the relative size difference of respective dimensions. Subsequently, the determined coordinates of both sub-packages were transformed to fit the coordinates of the extracted cubes. This could be achieved using the 18 selected x,y, and z coordinates as subtrahends for the determined coordinates of both sub-packages. All coordinates within the boundaries of the 60µm^3^ cubes with a tolerance of ± 5µm were selected for cell detection assessment and stored in xml files. The image cubes were visualized in Napari with the option to switch between 2-dimensional (2D) stacks and 3-dimensional (3D) visualization. Two human experts were counting cells in 2D stacks first. Subsequently the experts switched to 3D space to filter for redundantly counted cells in the previous step. After the human experts finished counting the transformed coordinates of ClearFinder’s sub-packages were opened in Napari and the cell counting was assessed in 3D space

Supplementary material 4: Figure 4. Cell detection performance assessment of tools compared to human experts. **A** Venn diagram of absolute cell counts of human experts from 3 × 6 randomly selected image cubes with a volume of 60 µm^3^ in samples #1, #2 and #3. **B**, **C** Venn diagram of cells counted by both human experts and the respective tool in the selected image cubes. **D** Boxplot showing the cell count ratio between cells detected by the respective tool and cells detected by both human experts. **E** Boxplot showing the cell count ratio between cells detected by the respective tool and cells detected by both human experts in the respective brain samples

Supplementary material 5 Embedded ontolo gy files for #1, #2, #3 analyzed with ClearMap and CellFinder

Supplementary material 6 Parameters of cell detection used for analysis of data

Supplementary material 7 Screenshots from ClearMap sub-package of ClearFinder

Supplementary material 8 Screenshots from CellFinder sub-package of ClearFinder

## Data Availability

Project name: ClearFinder. Project home page: https://github.com/stegiopast/ClearFinder . Operating system(s): Linux. Programming language: Python. Other requirements: Processors: 20 (Threads), RAM: 128 GB. License: GNU General Public License v3.0. Any restrictions to use by non-academics: license required. Cell counts data is provided within the supplementary information files.

## Abbreviations

DBE: Dibenzyl ether
DCM: Dichloromethane
DMSO: Dimethyl sulfoxide
GFP: Green fluorescent protein
GUI: Graphical user interface
i.p.: Intraperitoneal
IEG: Immediate-early genes
NDS: Normal donkey serum
PBS: Phosphate-buffered saline
PCA: Principal component analysis
PFA: Paraformaldehyde
PTx0.5: PBS with 0.5% TritonX-100
PTwHx0.5: PBS with 0.5% Tween 20 and 10 mg/ml heparin
RI: Refractive index
RT: Room temperature

## References

[1] DeNardo L, Luo L. Genetic strategies to access activated neurons. Curr Opin Neurobiol. 2017;45:121–9. 10.1016/j.conb.2017.05.014. 28577429 10.1016/j.conb.2017.05.014 PMC5810937

[2] Ueda HR, Erturk A, Chung K, Gradinaru V, Chedotal A, Tomancak P, et al. Tissue clearing and its applications in neuroscience. Nat Rev Neurosci. 2020;21:61–79. 10.1038/s41583-019-0250-1. 32152524 10.1038/s41583-020-0291-5

[3] Renier N, Adams EL, Kirst C, Wu Z, Azevedo R, Kohl J, et al. Mapping of brain activity by automated volume analysis of immediate early genes. Cell. 2016;165:1789–802. 10.1016/j.cell.2016.05.007. 27238021 10.1016/j.cell.2016.05.007 PMC4912438

[4] Kirst C, Skriabine S, Vieites-Prado A, Topilko T, Bertin P, Gerschenfeld G, et al. Mapping the fine-scale organization and plasticity of the brain vasculature. Cell. 2020;180:780-795.e25. 10.1016/j.cell.2020.01.028. 32059781 10.1016/j.cell.2020.01.028

[5] Tyson AL, Rousseau CV, Niedworok CJ, Keshavarzi S, Tsitoura C, Cossell L, et al. A deep learning algorithm for 3D cell detection in whole mouse brain image datasets. Plos Comput Biol. 2021;17:e1009074. 10.1371/journal.pcbi.1009074. 34048426 10.1371/journal.pcbi.1009074 PMC8191998

[6] Negwer M, Bosch B, Bormann M, Hesen R, Lütje L, Aarts L, et al. FriendlyClearMap: an optimized toolkit for mouse brain mapping and analysis. GigaScience. 2023;12:giad035. 10.1093/gigascience/giad035. 10.1093/gigascience/giad035 PMC10205001 37222748

[7] Denny CA, Kheirbek MA, Alba EL, Tanaka KF, Brachman RA, Laughman KB, et al. Hippocampal memory traces are differentially modulated by experience, time, and adult neurogenesis. Neuron. 2014;83:189–201. 10.1016/j.neuron.2014.05.018. 24991962 10.1016/j.neuron.2014.05.018 PMC4169172

[8] Mo A, Mukamel EA, Davis FP, Luo C, Henry GL, Picard S, et al. Epigenomic signatures of neuronal diversity in the mammalian brain. Neuron. 2015;86:1369–84. 10.1016/j.neuron.2015.05.018. 26087164 10.1016/j.neuron.2015.05.018 PMC4499463

[9] Butto T, Chongtham MC, Mungikar K, Hartwich D, Linke M, Ruffini N, et al. Characterization of transcriptional profiles associated with stress-induced neuronal activation in Arc-GFP mice. Mol Psychiatry. 2024. 10.1038/s41380-024-02555-z. 38649752 10.1038/s41380-024-02555-z PMC11449785

[10] Jin M, Nguyen JD, Weber SJ, Mejias-Aponte CA, Madangopal R, Golden SA. SMART: an open-source extension of wholebrain for intact mouse brain registration and segmentation. 2022. ENeuro. 10.1523/eneuro.0482-21.2022. 10.1523/ENEURO.0482-21.2022 PMC9070730 35396258

[11] Sofroniew N, Lambert T, Evans K, Nunez-Iglesias J, Bokota G, Winston P, et al. napari: a multi-dimensional image viewer for Python (v0.4.17rc8). 2022. Zenodo. 10.5281/zenodo.7276432.

[12] Tommaso PD, Chatzou M, Floden EW, Barja PP, Palumbo E, Notredame C. Nextflow enables reproducible computational workflows. Nat Biotechnol. 2017;35:316–9. 10.1038/nbt.3820. 28398311 10.1038/nbt.3820

[13] Maza E, Frasse P, Senin P, Bouzayen M, Zouine M. Comparison of normalization methods for differential gene expression analysis in RNA-Seq experiments. Commun Integr Biol. 2013;6:e25849. 10.4161/cib.25849. 26442135 10.4161/cib.25849 PMC3918003

[14] Kimbrough A, Kallupi M, Smith LC, Simpson S, Collazo A, George O. Characterization of the brain functional architecture of psychostimulant withdrawal using single-cell whole-brain imaging. Eneuro. 2021. 10.1523/eneuro.0208-19.2021. 34580158 10.1523/ENEURO.0208-19.2021 PMC8570684

[15] Roland AV, Coelho CAO, Haun HL, Gianessi CA, Lopez MF, D’Ambrosio S, et al. Alcohol dependence modifies brain networks activated during withdrawal and reaccess: a c-Fos–based analysis in mice. Biol Psychiatry. 2023;94:393–404. 10.1016/j.biopsych.2023.01.018. 36736419 10.1016/j.biopsych.2023.01.018 PMC10517410

[16] Liebmann T, Renier N, Bettayeb K, Greengard P, Tessier-Lavigne M, Flajolet M. Three-dimensional study of Alzheimer’s disease hallmarks using the iDISCO clearing method. Cell Rep. 2016;16:1138–52. 10.1016/j.celrep.2016.06.060. 27425620 10.1016/j.celrep.2016.06.060 PMC5040352

[17] Detrez JR, Maurin H, Kolen KV, Willems R, Colombelli J, Lechat B, et al. Regional vulnerability and spreading of hyperphosphorylated tau in seeded mouse brain. Neurobiol Dis. 2019;127:398–409. 10.1016/j.nbd.2019.03.010. 30878534 10.1016/j.nbd.2019.03.010

[18] Madangopal R, Szelenyi ER, Nguyen J, Brenner MB, Drake OR, Pham DQ, et al. Incubation of palatable food craving is associated with brain-wide neuronal activation in mice. Proc Natl Acad Sci. 2022;119:e2209382119. 10.1073/pnas.2209382119. 36603188 10.1073/pnas.2209382119 PMC9659381

[19] Wang Q, Ding S-L, Li Y, Royall J, Feng D, Lesnar P, et al. The Allen mouse brain common coordinate framework: a 3D reference atlas. Cell. 2020;181:936-953.e20. 10.1016/j.cell.2020.04.007. 32386544 10.1016/j.cell.2020.04.007 PMC8152789

