# Supplementary figures and images for: ClearFinder: a Python GUI for annotating cells in cleared mouse brain
Source: BMC Bioinformatics. 2025 Jan 21;26:24. doi: 10.1186/s12859-025-06039-x (PMC11753021; doi:10.1186/s12859-025-06039-x)

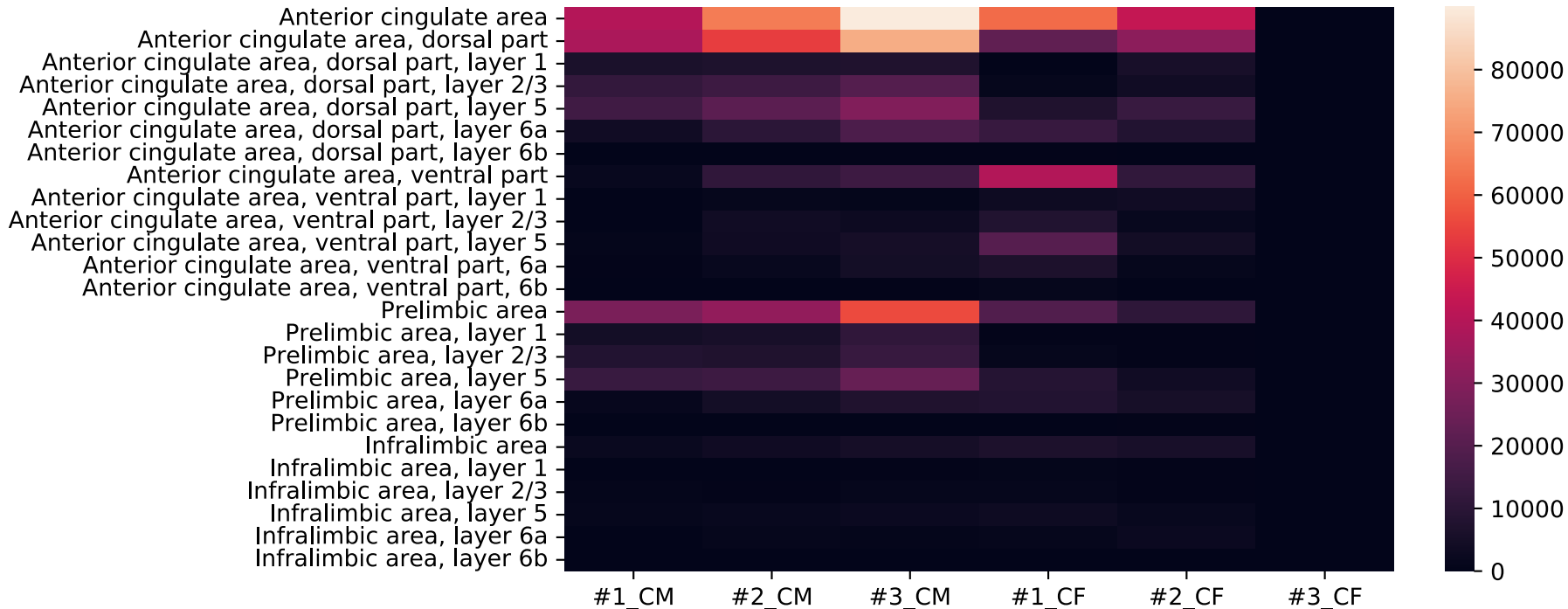

Supplement: Supplementary file 1 — Supplementary material 1: Figure 1. Cell detection efficiency in cortical regions. Heatmap indicating the number of cells detected in the subregions of the anterior cingulate, prelimbic, and infralimbic areas as detected in each sample by ClearMap (CM) and CellFinder (CF) using the same threshold. Color-bar corresponds to cell count. Regions with no cell counts were not accounted in the heatmap [file 12859_2025_6039_MOESM1_ESM.pdf]

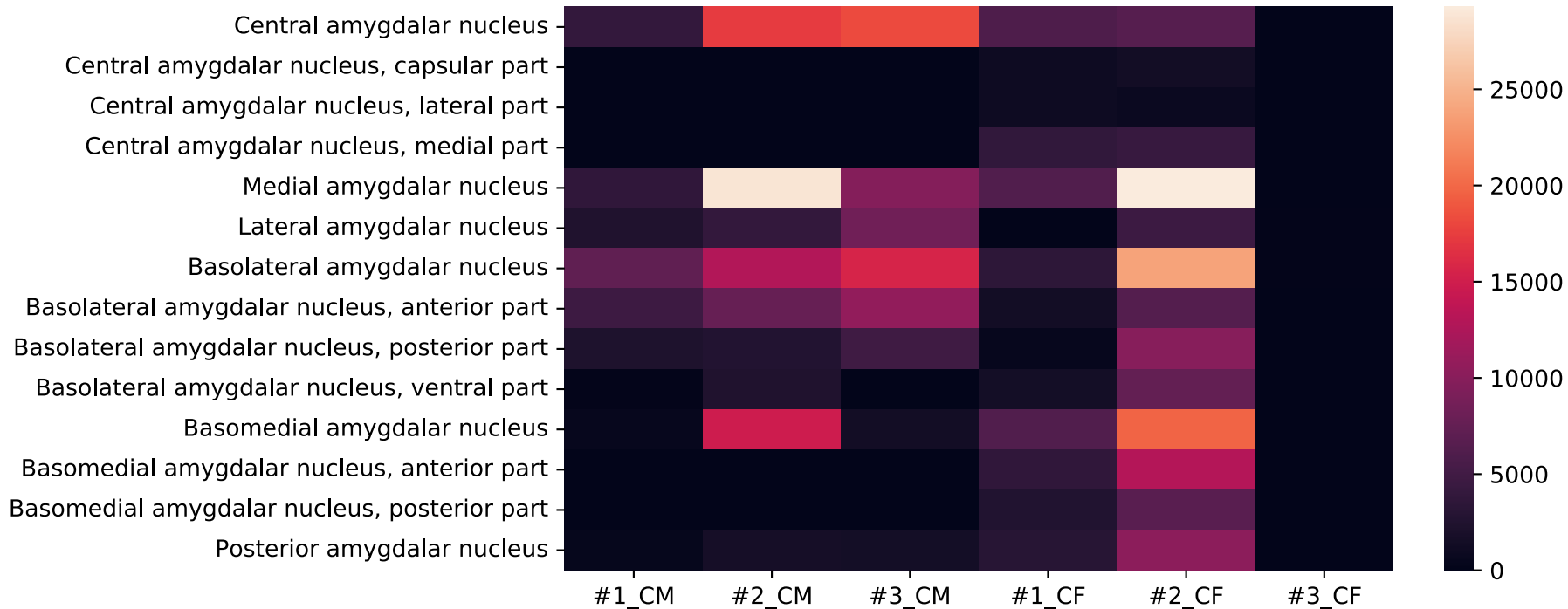

Supplement: Supplementary file 2 — Supplementary material 2: Figure 2. Cell detection efficiency in subcortical regions. Heatmap indicating the number of cells detected in the subregions of the central, medial, lateral, basolateral, basomedial, and posterior amygdalar nuclei as detected in each sample by ClearMap (CM) and CellFinder (CF) using the same threshold. Color-bar corresponds to cell count. Regions with no cell counts were not accounted in the heatmap [file 12859_2025_6039_MOESM2_ESM.pdf]

A

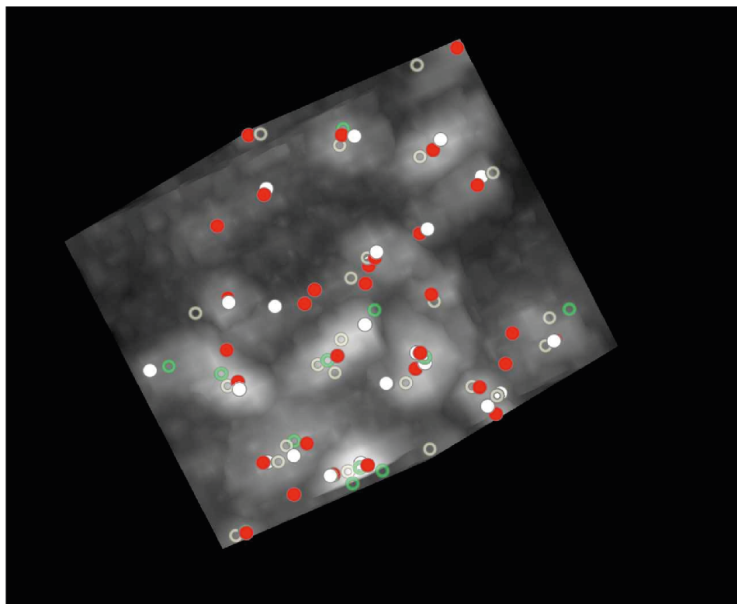

B

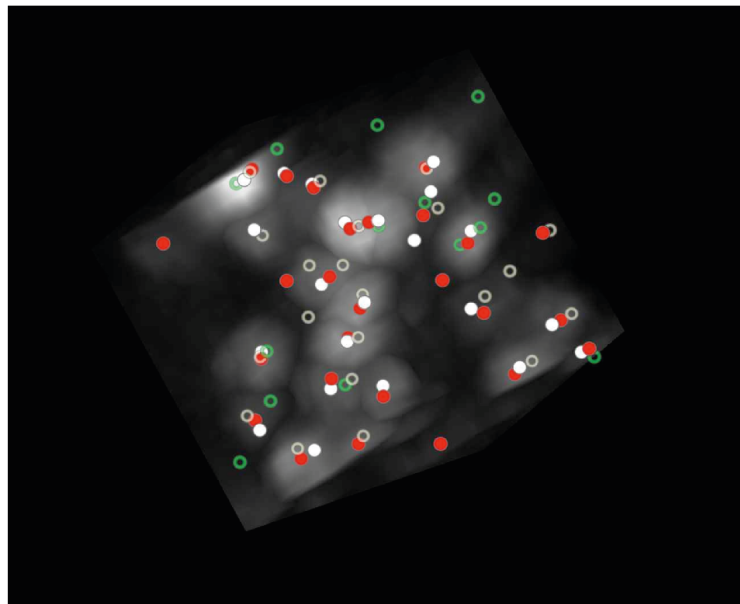

C

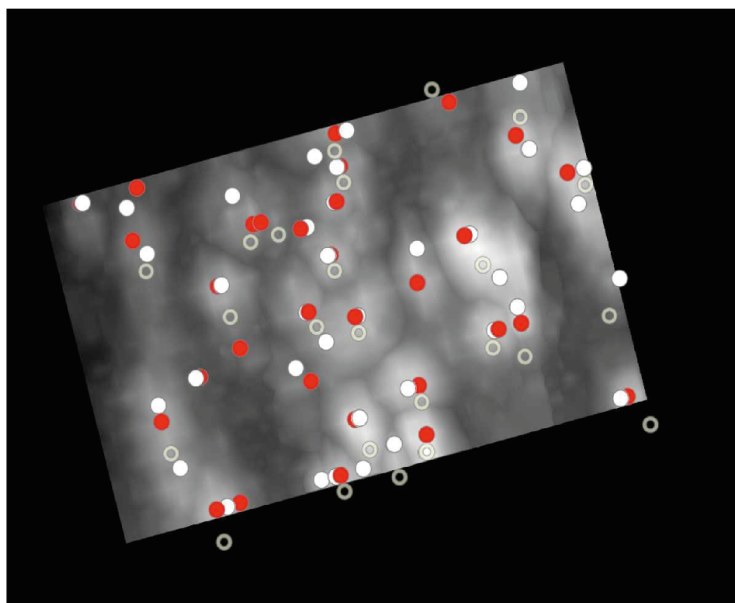

Supplement: Supplementary file 3 — Supplementary material 3: Figure 3. Image cubes for cell detection performance assessment. Examples of image cubes of sample #1 (A), #2 (B) and #3 (C). White dots mark counted cells of expert 1, red dots of expert 2, yellow circles of ClearMap and green circles of CellFinder. For sample collection 18 randomly selected x,y and z coordinates were generated. With these start coordinates cubes with a volume of 60µm3 of the raw signal files were created using ImageJ. Due to inevitable resizing events during the Cell detection process with CellFinder, the coordinates had to be re-transformed to the original signal image sizes. The latter was performed by multiplying the determined coordinates with the relative size difference of respective dimensions. Subsequently, the determined coordinates of both sub-packages were transformed to fit the coordinates of the extracted cubes. This could be achieved using the 18 selected x,y, and z coordinates as subtrahends for the determined coordinates of both sub-packages. All coordinates within the boundaries of the 60µm3 cubes with a tolerance of ± 5µm were selected for cell detection assessment and stored in xml files. The image cubes were visualized in Napari with the option to switch between 2-dimensional (2D) stacks and 3-dimensional (3D) visualization. Two human experts were counting cells in 2D stacks first. Subsequently the experts switched to 3D space to filter for redundantly counted cells in the previous step. After the human experts finished counting the transformed coordinates of ClearFinder’s sub-packages were opened in Napari and the cell counting was assessed in 3D space [file 12859_2025_6039_MOESM3_ESM.pdf]

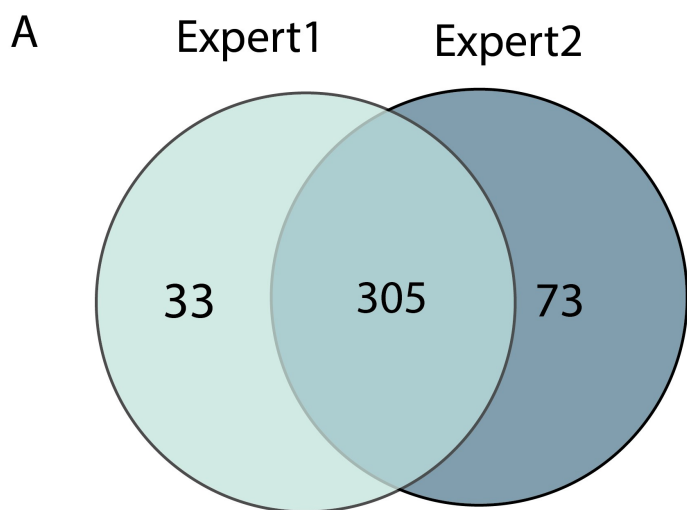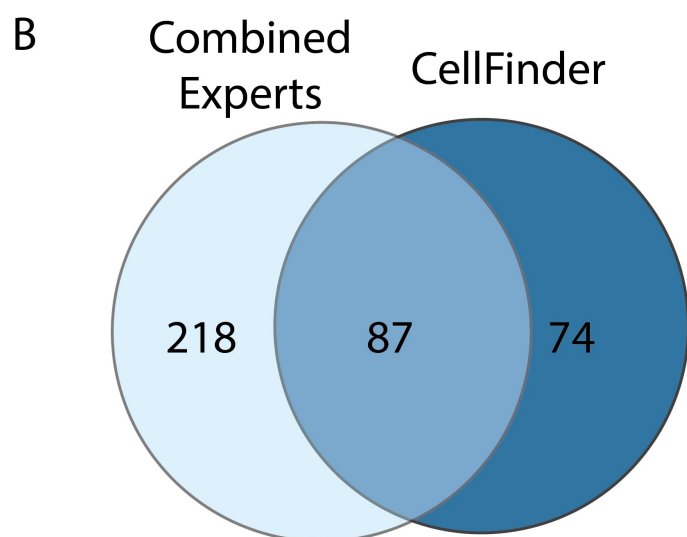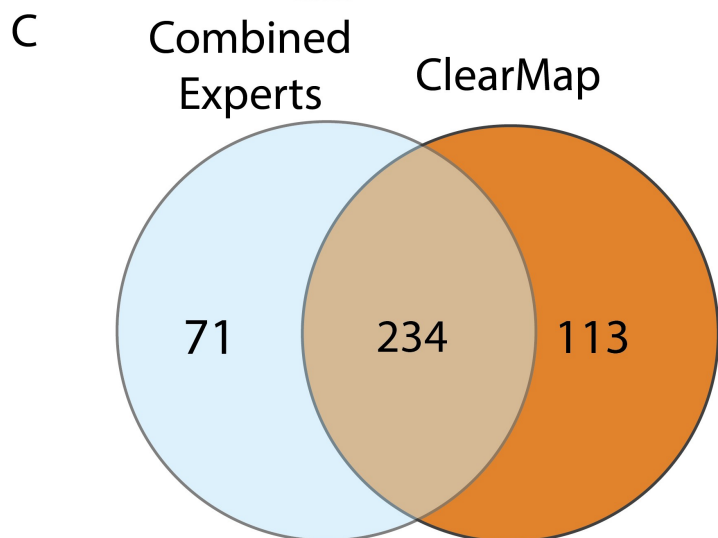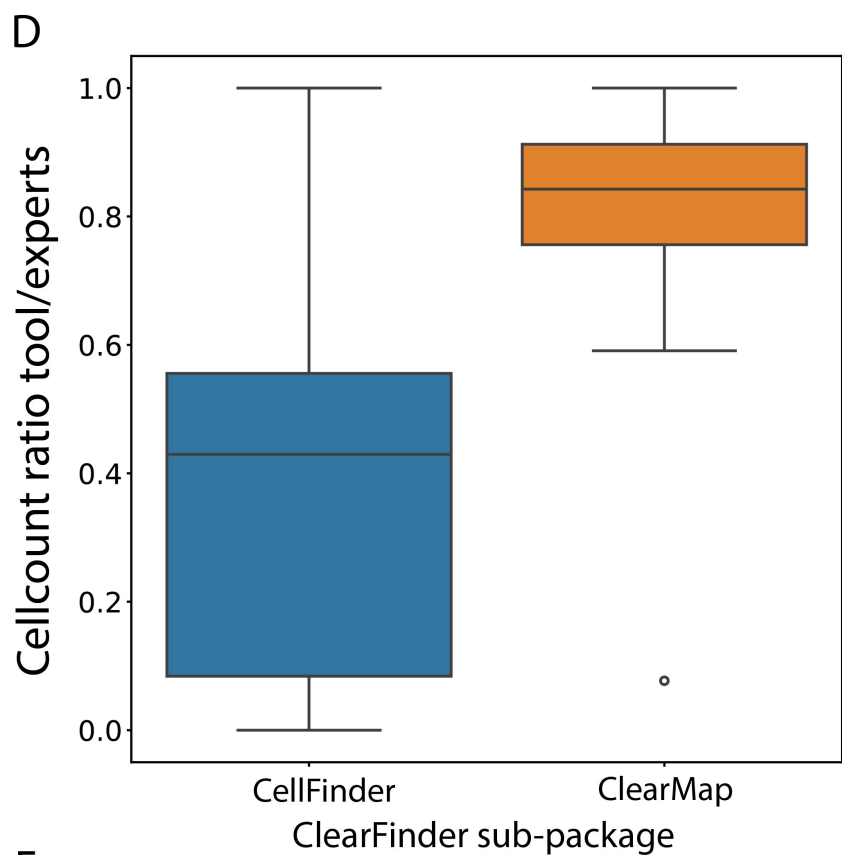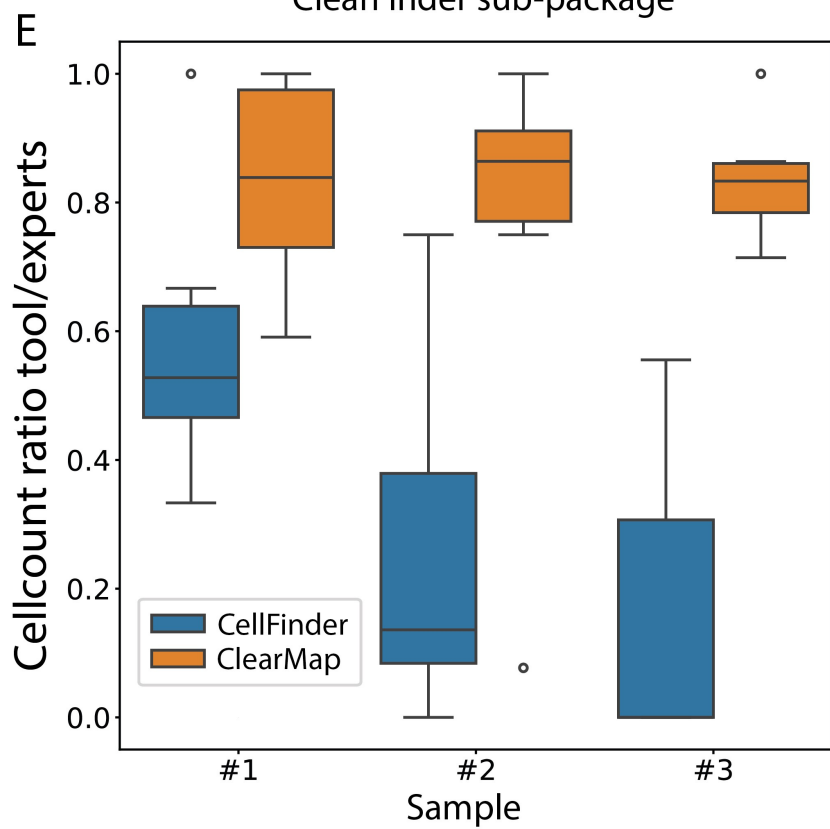

Supplement: Supplementary file 4 — Supplementary material 4: Figure 4. Cell detection performance assessment of tools compared to human experts. A Venn diagram of absolute cell counts of human experts from 3 × 6 randomly selected image cubes with a volume of 60 µm3 in samples #1, #2 and #3. B, C Venn diagram of cells counted by both human experts and the respective tool in the selected image cubes. D Boxplot showing the cell count ratio between cells detected by the respective tool and cells detected by both human experts. E Boxplot showing the cell count ratio between cells detected by the respective tool and cells detected by both human experts in the respective brain samples [file 12859_2025_6039_MOESM4_ESM.pdf]
