# Supplementary material for: ClearFinder: a Python GUI for annotating cells in cleared mouse brain
Source: BMC Bioinformatics. 2025 Jan 21;26:24. doi: 10.1186/s12859-025-06039-x (PMC11753021; doi:10.1186/s12859-025-06039-x)
Supplement: Supplementary file 7 — Supplementary material 7 Screenshots from ClearMap sub-package of ClearFinder [file 12859_2025_6039_MOESM7_ESM.pdf]

ClearMap2

Determine Path | Rename Path | Resampling | Alignment | Cell Detection | Grouping and Normalization | Analysis and Plots

Set Workspace:

Input path of interest: [ ] Pick Workspace Directory | C01 | Hemisphere | Set workspace | Rename files in Auto | Rename files in Signal C01 | Rename files in Signal C02

Testdata option:

Make Testdata | Border region

Test Mode

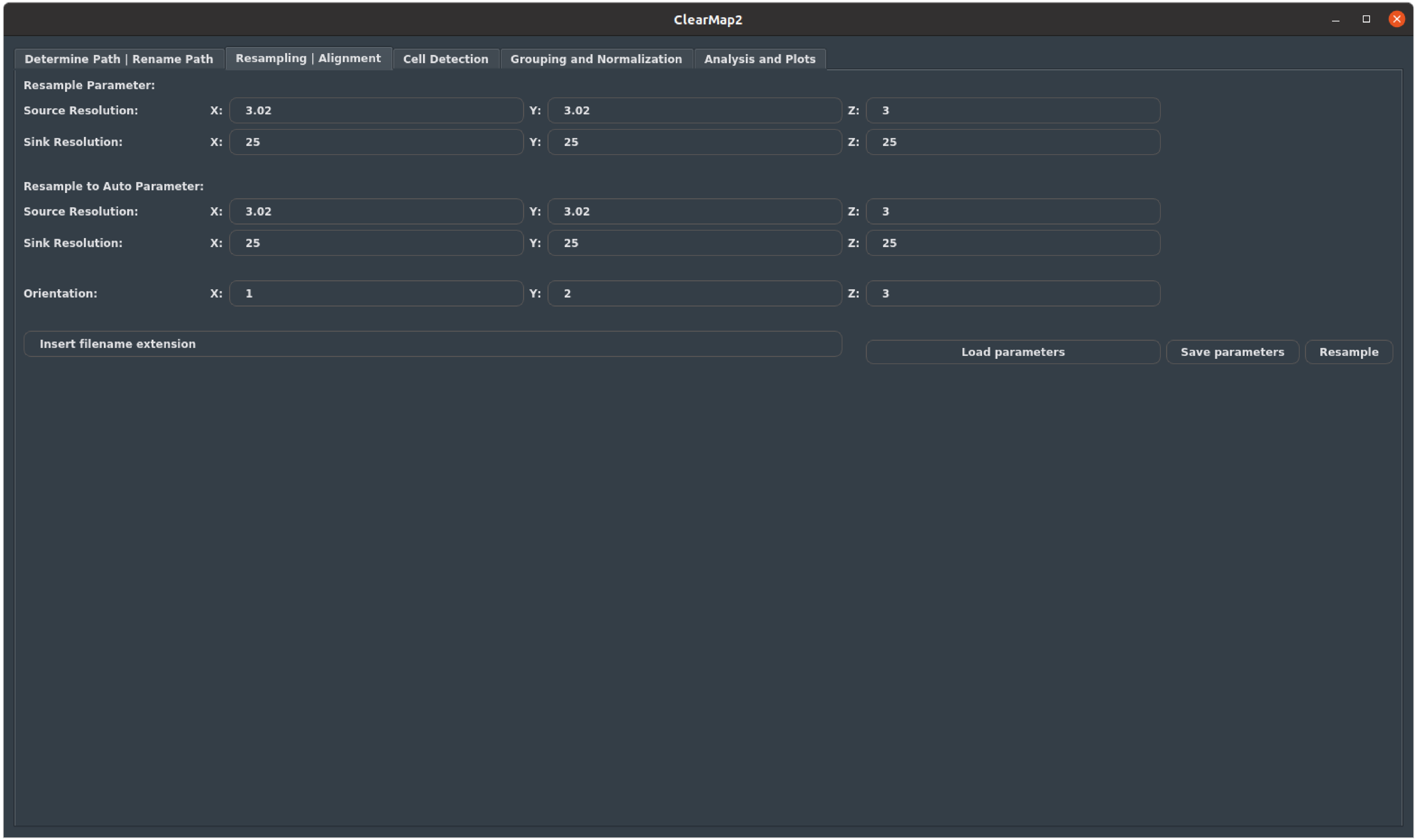
ClearMap2
Determine Path | Rename Path
Resampling | Alignment
Cell Detection
Grouping and Normalization
Analysis and Plots
Resample Parameter:
Source Resolution: X: 3.02 Y: 3.02 Z: 3
Sink Resolution: X: 25 Y: 25 Z: 25
Resample to Auto Parameter:
Source Resolution: X: 3.02 Y: 3.02 Z: 3
Sink Resolution: X: 25 Y: 25 Z: 25
Orientation: X: 1 Y: 2 Z: 3
Insert filename extension
Load parameters
Save parameters
Resample

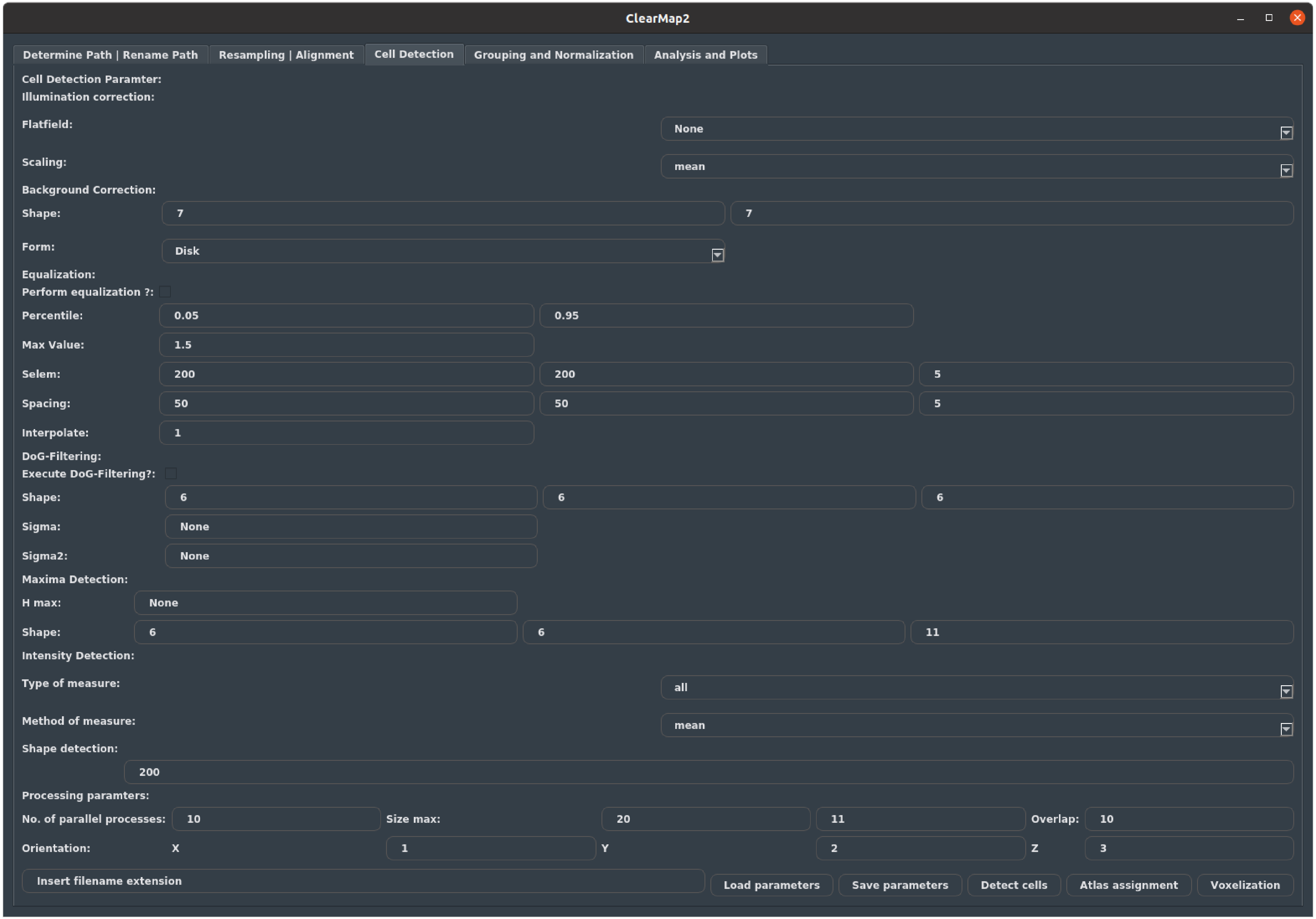
ClearMap2
Determine Path | Rename Path
Resampling | Alignment
Cell Detection
Grouping and Normalization
Analysis and Plots
Cell Detection Paramter:
Illumination correction:
Flatfield:
None
Scaling:
mean
Background Correction:
Shape:
7
7
Form:
Disk
Equalization:
Perform equalization ?:
Percentile:
0.05
0.95
Max Value:
1.5
Selem:
200
200
5
Spacing:
50
50
5
Interpolate:
1
DoG-Filtering:
Execute DoG-Filtering?:
Shape:
6
6
6
Sigma:
None
Sigma2:
None
Maxima Detection:
H max:
None
Shape:
6
6
11
Intensity Detection:
Type of measure:
all
Method of measure:
mean
Shape detection:
200
Processing paramters:
No. of parallel processes:
10
Size max:
20
11
Overlap:
10
Orientation:
X
1
Y
2
Z
3
Insert filename extension
Load parameters
Save parameters
Detect cells
Atlas assignment
Voxelization

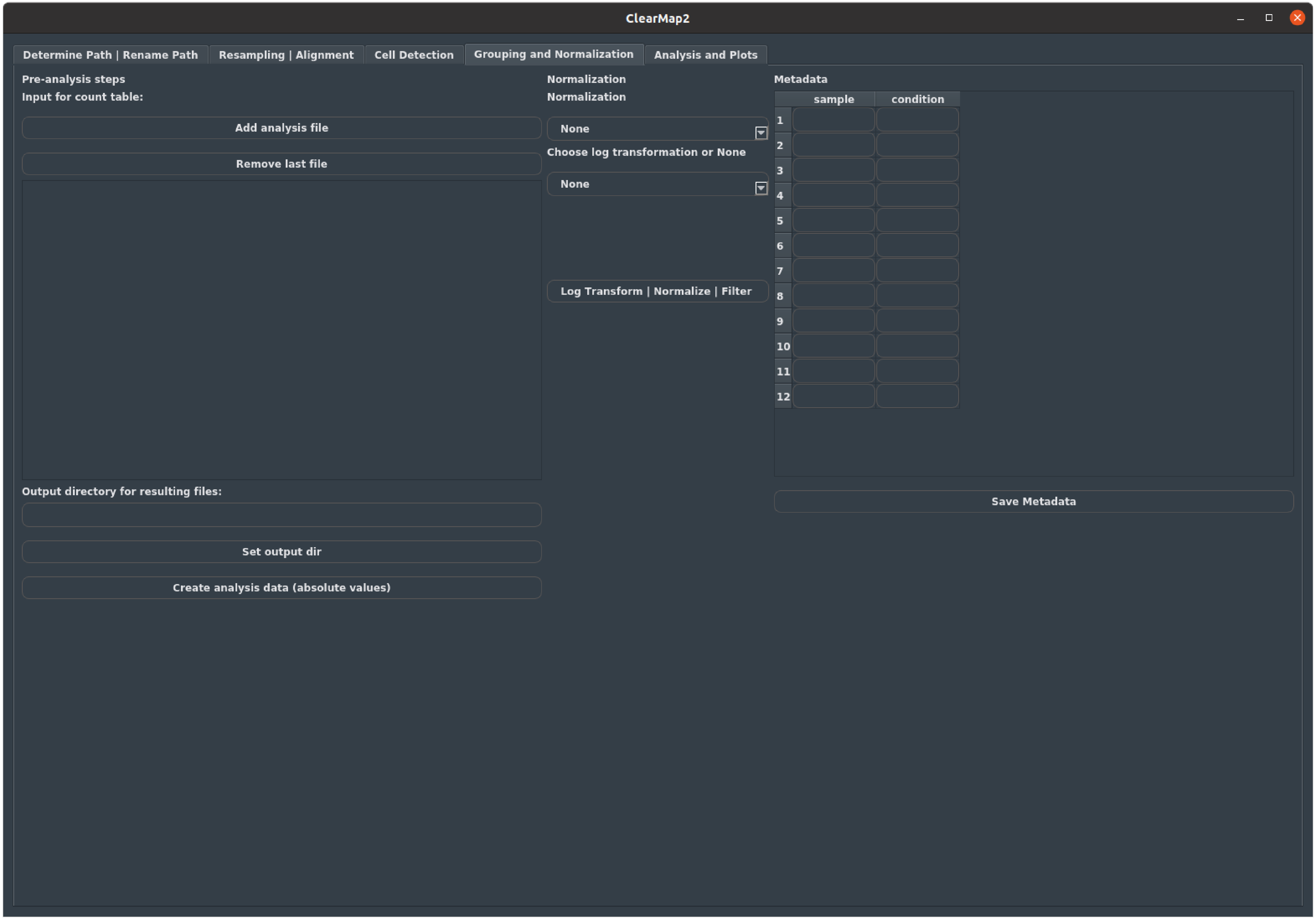
ClearMap2
Determine Path | Rename Path
Resampling | Alignment
Cell Detection
Grouping and Normalization
Analysis and Plots
Pre-analysis steps
Input for count table:
Add analysis file
Remove last file
Output directory for resulting files:
Set output dir
Create analysis data (absolute values)
Normalization
Normalization
None
Choose log transformation or None
None
Log Transform | Normalize | Filter
Metadata
sample
condition
1
2
3
4
5
6
7
8
9
10
11
12
Save Metadata

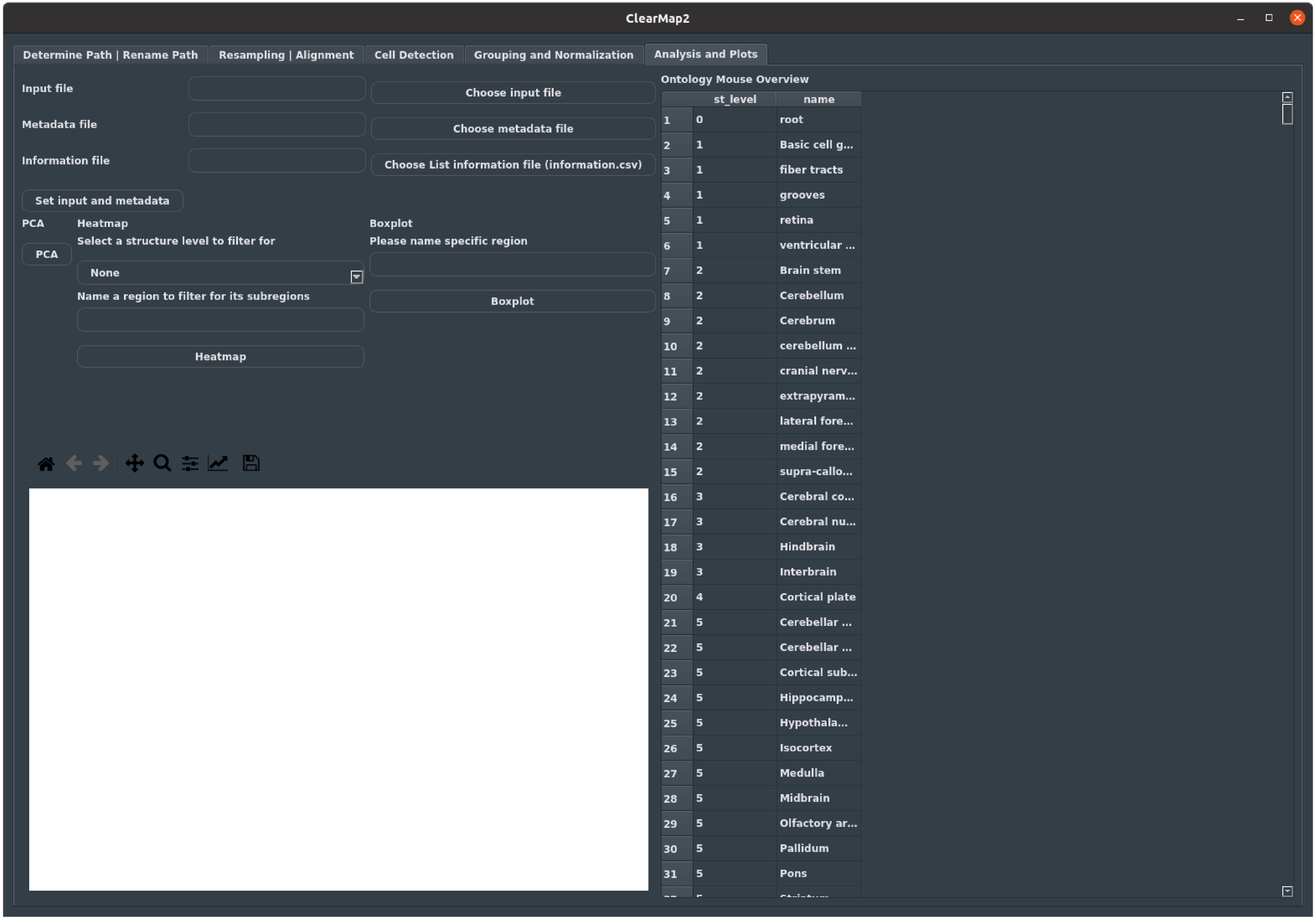
ClearMap2
Determine Path | Rename Path
Resampling | Alignment
Cell Detection
Grouping and Normalization
Analysis and Plots
Input file
Choose input file
Metadata file
Choose metadata file
Information file
Choose List information file (information.csv)
Set input and metadata
PCA
PCA
Heatmap
Select a structure level to filter for
None
Name a region to filter for its subregions
Heatmap
Boxplot
Please name specific region
Boxplot
Ontology Mouse Overview
st_level
name
1 0 root
2 1 Basic cell g...
3 1 fiber tracts
4 1 grooves
5 1 retina
6 1 ventricular ...
7 2 Brain stem
8 2 Cerebellum
9 2 Cerebrum
10 2 cerebellum ...
11 2 cranial nerv...
12 2 extrapyram...
13 2 lateral fore...
14 2 medial fore...
15 2 supra-callo...
16 3 Cerebral co...
17 3 Cerebral nu...
18 3 Hindbrain
19 3 Interbrain
20 4 Cortical plate
21 5 Cerebellar ...
22 5 Cerebellar ...
23 5 Cortical sub...
24 5 Hippocamp...
25 5 Hypothala...
26 5 Isocortex
27 5 Medulla
28 5 Midbrain
29 5 Olfactory ar...
30 5 Pallidum
31 5 Pons
