# Supplementary material for: ClearFinder: a Python GUI for annotating cells in cleared mouse brain
Source: BMC Bioinformatics. 2025 Jan 21;26:24. doi: 10.1186/s12859-025-06039-x (PMC11753021; doi:10.1186/s12859-025-06039-x)
Supplement: Supplementary file 8 — Supplementary material 8 Screenshots from CellFinder sub-package of ClearFinder [file 12859_2025_6039_MOESM8_ESM.pdf]

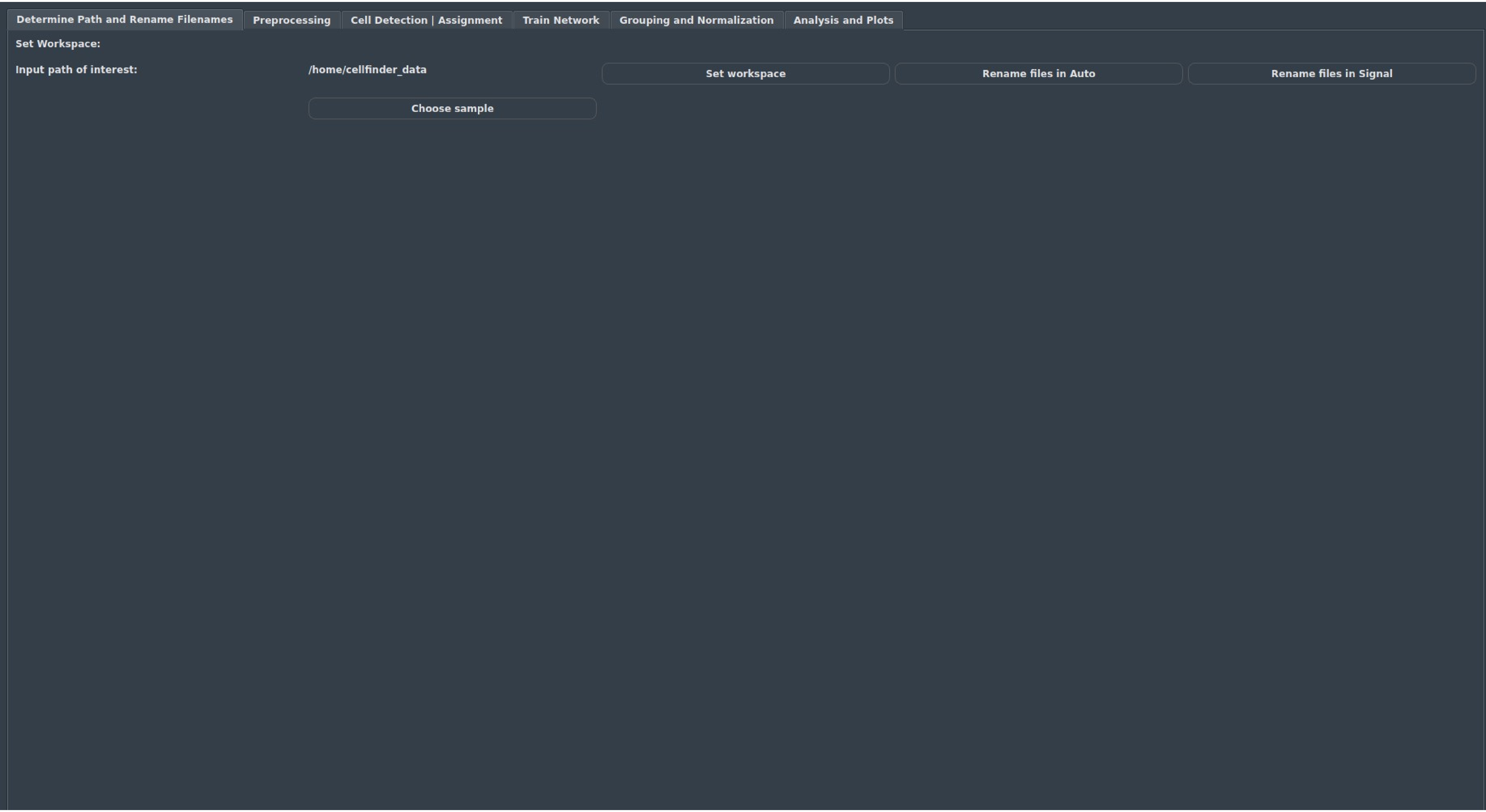

Determine Path and Rename Filenames
Preprocessing
Cell Detection | Assignment
Train Network
Grouping and Normalization
Analysis and Plots
Set Workspace:
Input path of interest:
/home/cellfinder_data
Set workspace
Rename files in Auto
Rename files in Signal
Choose sample

Determine Path and Rename Filenames | Preprocessing | Cell Detection | Assignment | Train Network | Grouping and Normalization | Analysis and Plots

Insert voxel sizes:

Voxel size Signal X:	5.00
Voxel size Signal Y:	2.00
Voxel Size Signal Z:	2.00
Voxel size Auto X:	5.00
Voxel size Auto Y:	2.00
Voxel Size Auto Z:	2.00

Start Preprocessing

Determine Path and Rename Filenames | Preprocessing | Cell Detection | Assignment | Train Network | Grouping and Normalization | Analysis and Plots

Number of cpus available:	4
Lower Boundary measured in number of standarddeviations above mean illumination:	10
Mean soma diamter:	16
Mean cell size in xy plane:	6
Mean cell size in z plane:	6
Gaussian Filter:	0.2
Custom pretrained model:	Choose model
Choose brain orientation (anterior/posterior,superior/inferior,left/right)	asl

Insert filename extension | Load parameters | Safe parameters | Start Cell Detection

Whole brain | Embed Ontology

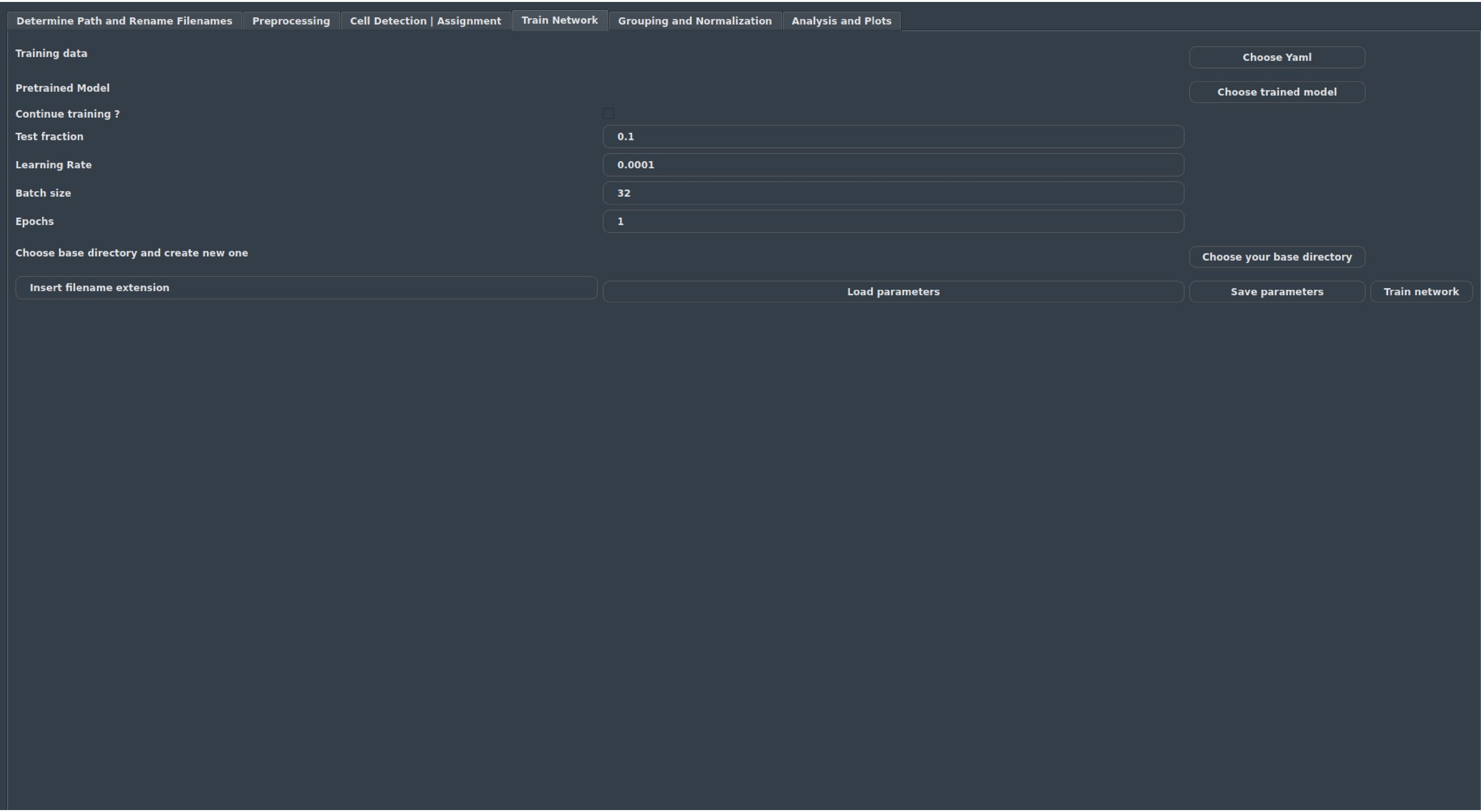
Determine Path and Rename Filenames
Preprocessing
Cell Detection | Assignment
Train Network
Grouping and Normalization
Analysis and Plots
Training data
Choose Yaml
Pretrained Model
Choose trained model
Continue training ?
Test fraction
0.1
Learning Rate
0.0001
Batch size
32
Epochs
1
Choose base directory and create new one
Choose your base directory
Insert filename extension
Load parameters
Save parameters
Train network

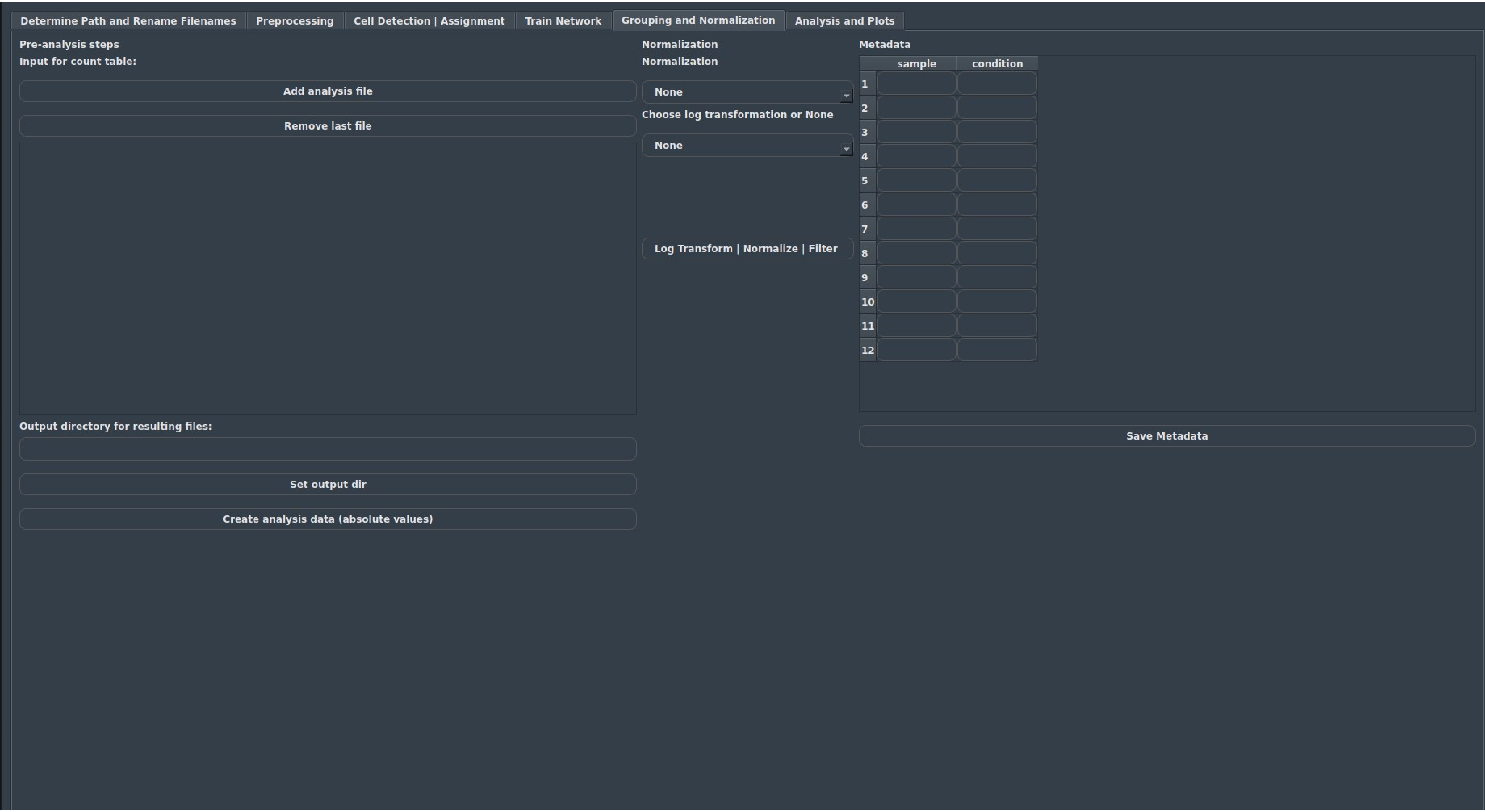
Determine Path and Rename Filenames
Preprocessing
Cell Detection | Assignment
Train Network
Grouping and Normalization
Analysis and Plots
Pre-analysis steps
Input for count table:
Add analysis file
Remove last file
Output directory for resulting files:
Set output dir
Create analysis data (absolute values)
Normalization
Normalization
None
Choose log transformation or None
None
Log Transform | Normalize | Filter
Metadata
sample
condition
1
2
3
4
5
6
7
8
9
10
11
12
Save Metadata

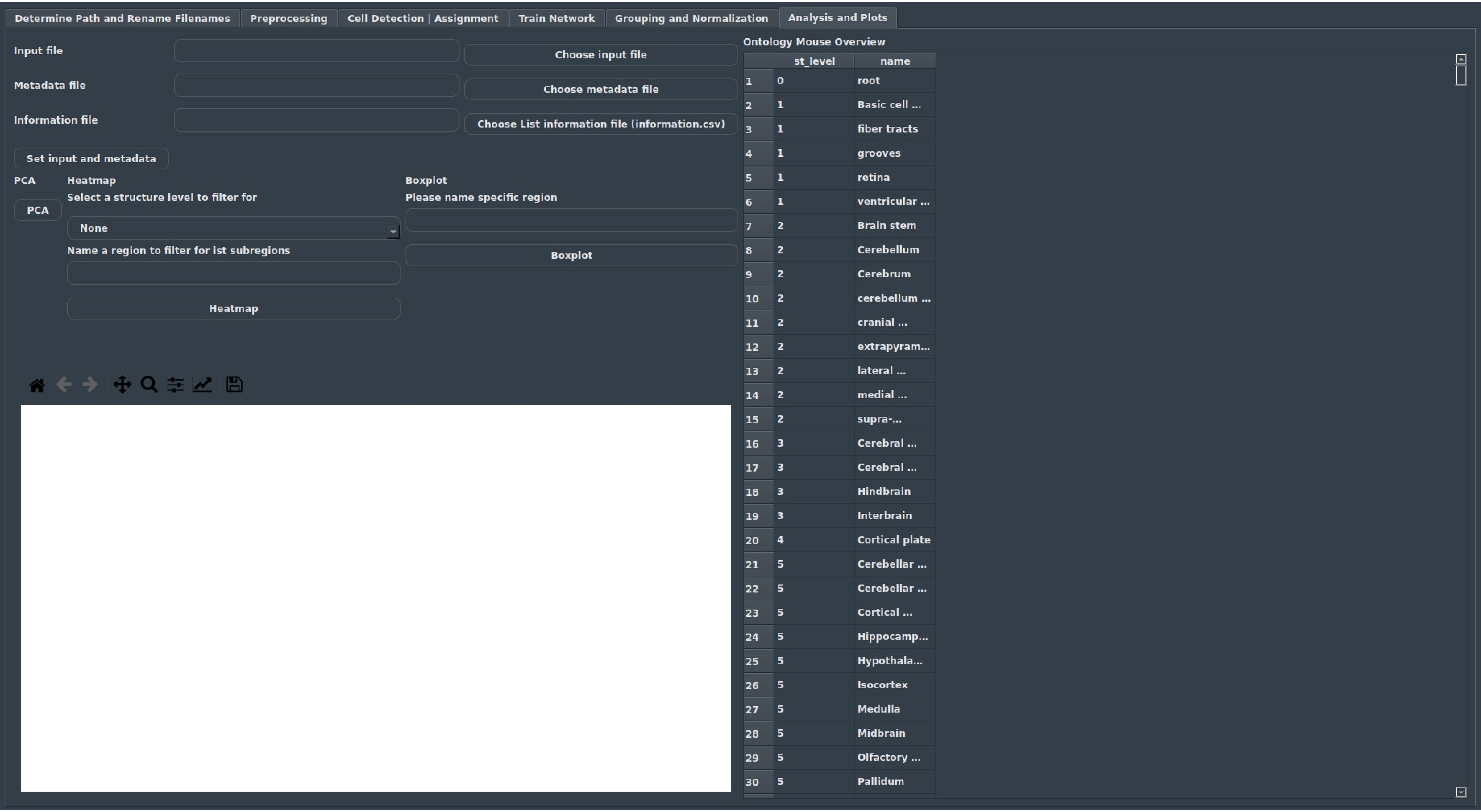
Determine Path and Rename Filenames
Preprocessing
Cell Detection | Assignment
Train Network
Grouping and Normalization
Analysis and Plots
Input file
Choose input file
Metadata file
Choose metadata file
Information file
Choose List information file (information.csv)
Set input and metadata
PCA
Heatmap
Boxplot
Select a structure level to filter for
Please name specific region
PCA
None
Name a region to filter for ist subregions
Boxplot
Heatmap
Ontology Mouse Overview
st_level name
1 0 root
2 1 Basic cell ...
3 1 fiber tracts
4 1 grooves
5 1 retina
6 1 ventricular ...
7 2 Brain stem
8 2 Cerebellum
9 2 Cerebrum
10 2 cerebellum ...
11 2 cranial ...
12 2 extrapyram...
13 2 lateral ...
14 2 medial ...
15 2 supra-...
16 3 Cerebral ...
17 3 Cerebral ...
18 3 Hindbrain
19 3 Interbrain
20 4 Cortical plate
21 5 Cerebellar ...
22 5 Cerebellar ...
23 5 Cortical ...
24 5 Hippocamp...
25 5 Hypothala...
26 5 Isocortex
27 5 Medulla
28 5 Midbrain
29 5 Olfactory ...
30 5 Pallidum
